# US cardiovascular mortality before and during COVID-19: CDC WONDER vs GBD comparison

**DOI:** 10.1093/aje/kwag153

**Published:** 2026-07-08

**Authors:** Mirza Mohammad Ali Baig, Muhammad Umar, Carol Wright Becker, Salim S Virani, Dmitry Abramov, Xiaoming Jia, Abdul Mannan Khan Minhas

**Affiliations:** Islamic International Medical College, Riphah International University, Rawalpindi, Pakistan; Global Burden of Diseases, IHME, University of Washington, Seattle, WA,, United States; Global Burden of Diseases, IHME, University of Washington, Seattle, WA,, United States; Department of Medicine, Khairpur Medical College, Khairpur Mir's, Pakistan; Department of Emergency Medicine, West Virginia University, Morgantown, WV, United States; Section of Cardiology, Department of Medicine, Aga Khan University, Karachi, Pakistan; Department of Population Health, Aga Khan University, Nairobi, Kenya; Division of Cardiovascular Medicine, Loma Linda University Medical Center, Loma Linda, CA, United States; Section of Cardiology, Department of Medicine, Baylor College of Medicine, Houston, TX, United States; Section of Cardiology, Department of Medicine, Baylor College of Medicine, Houston, TX, United States

## Introduction

Accurate national surveillance of cardiovascular disease (CVD) mortality is essential for informing prevention strategies and tracking progress toward US health goals.^1^ Two widely used sources—the Centers for Disease Control and Prevention Wide-Ranging Online Data for Epidemiologic Research (CDC WONDER) and the Global Burden of Disease (GBD) study—may produce different estimates of mortality due to their differing methodologies. CDC Wide-Ranging Online Data for Epidemiologic Research classifies causes of death based on the diagnoses entered on the death certificate (reported according to ICD codes), while GBD expands on International Classification of Diseases (ICD) codes with modeling techniques and reallocates ill-defined (“garbage”) codes to specific causes of death.^2^^,^^3^ Although both systems are commonly used for research and policy evaluations, the comparison of their estimates regarding US CVD mortality estimates has been limited. This comparison is particularly important now, as the COVID-19 pandemic disrupted death certification practices and altered CVD mortality trends, potentially widening discrepancies between data systems. Evaluating these differences in the current era provides timely insight into the reliability and interpretation of national CVD mortality estimates, and we therefore sought to evaluate potential effects of the pandemic on CVD mortality.

## Methods

We analyzed US CVD mortality from 1999-2023 using the GBD 2023 and CDC WONDER underlying cause-of-death databases. CDC Wide-Ranging Online Data for Epidemiologic Research data are compiled by the National Center of Health Statistics and contain data on more than 99% of deaths in the United States; it applies no redistribution of ill-defined codes and no statistical smoothing. Global Burden of Disease, by contrast, uses a multi-stage modeling framework. Among modeling approaches include systematic redistribution that reassigns deaths coded to non-specific or implausible underlying causes to specific causes, and the use of the cause of death ensemble modeling framework for further adjustment of mortality outputs. Full GBD methodological details are publicly available.^4-6^ To ensure comparability, we matched the same disease categories across both sources using explicit ICD-10 definitions. From the GBD 2023 cause hierarchy, we extracted deaths and crude mortality rates (CMRs) for: (1) total CVDs, (2) ischemic heart disease (IHD), (3) cerebrovascular disease (stroke), and (4) atrial fibrillation and flutter. Corresponding CDC WONDER estimates were obtained using underlying cause-of-death ICD-10 codes for each category: I00–I99 for total CVD, I20–I25 for ischemic heart disease, I60–I69 for stroke, and I48 for atrial fibrillation/flutter. Because GBD and CDC WONDER use different age-standardization approaches, we reported only absolute deaths and CMRs to avoid methodological bias. Percentage changes in deaths and CMRs from 1999 to 2023 were calculated for each disease category. In a pre-specified supplementary analysis, we examined all-cause mortality and 3 non-CVD cause categories: neoplasms, neurological diseases, and digestive diseases, which represent important contributors to established public health burden. Institutional review board approval was not required for this analysis of publicly available, de-identified data.

## Results

In 1999, GBD reported 918 092 cardiovascular deaths (CMR 329.8; 95% uncertainty interval [UI], 292.6-349.5), compared with 954 339 deaths (CMR 342.0; 95% CI, 341.3-342.7) in CDC WONDER. By 2023, these values were 900 759 (CMR 268.6; 95% UI, 233.3-288.5) and 915 973 deaths (CMR 273.5; 95% CI, 272.9-274.1), respectively. Overall, CMR declined by 18.56% in GBD and 20.03% in CDC WONDER. Category-specific trends showed mostly similar directions but notable magnitude gaps: IHD CMR decreased by 33.9% in GBD vs 45.0% in CDC WONDER, while stroke CMR declined by 11.1% and 19.0% from 1999 to 2023, respectively. In contrast, atrial fibrillation/flutter mortality increased in both, with larger gains in CDC (82.1%) compared with GBD (63.4%) from 1999 to 2023 (Tables S1 and S2). Percent differences in CVD reporting between GBD and CDC WONDER by year are shown in Figure 1.

**Figure 1:**
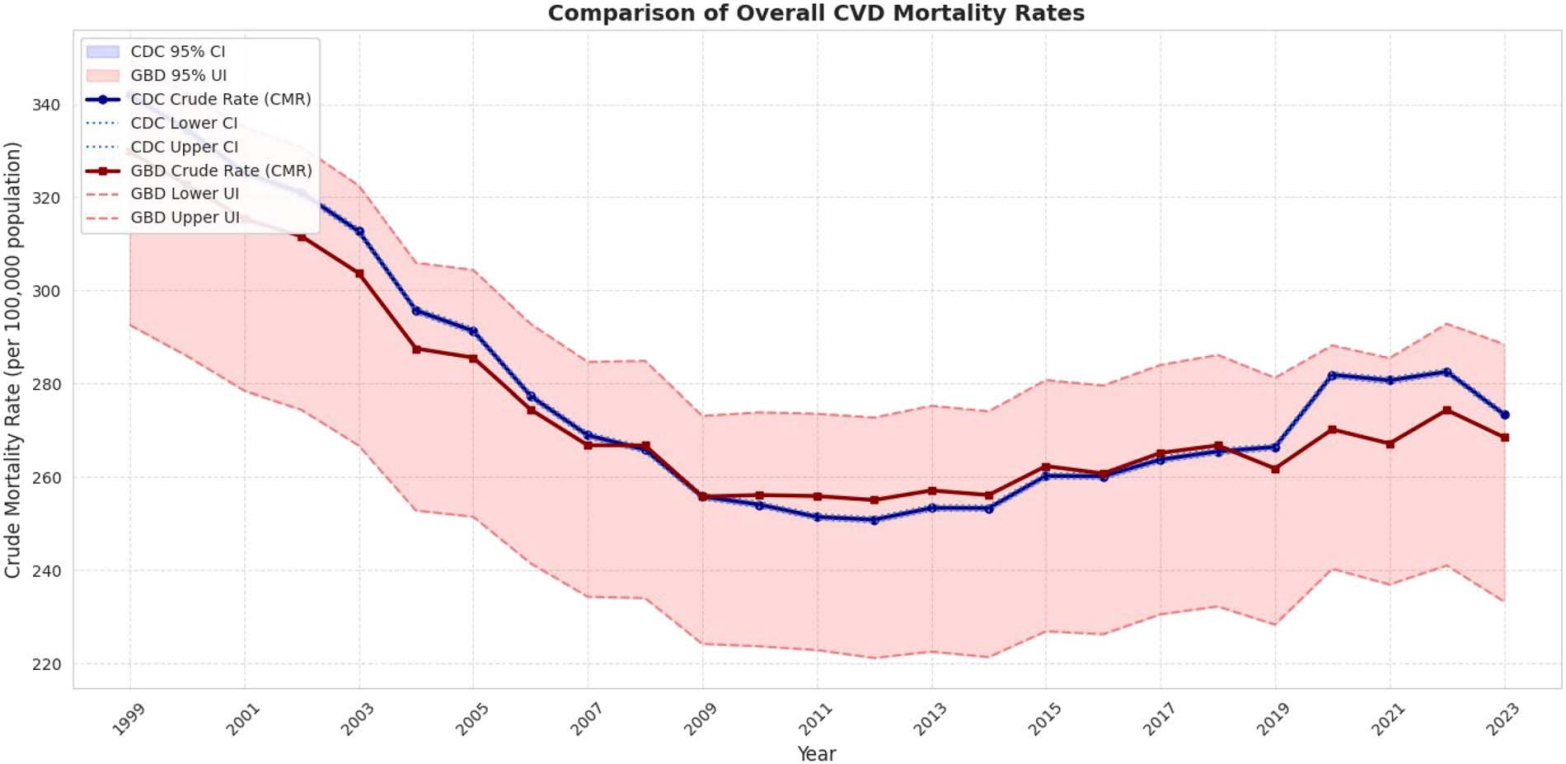
Comparison of cardiovascular disease (CVD) crude mortality rates (CMRs) per 100 000 population between CDC Wide-Ranging Online Data for Epidemiologic Research (CDC WONDER) and Global Burden of Disease (GBD; 1999-2023). The figure displays annual CMR estimates from CDC WONDER (solid line with 95% CIs) and GBD (solid line with 95% uncertainty intervals [UIs]) for total CVD mortality.

To assess whether database discrepancies extend beyond CVD, we conducted a supplementary analysis examining all-cause mortality and 3 non-CVD cause categories (digestive diseases, neoplasms, and neurological diseases) from 1999 to 2023. Detailed findings from the supplementary analyzes are presented in Table S3 and Figures S1-S4. For total CVD, CDC WONDER estimates fell within GBD’s 95% UI in all 25 years examined (1999-2023). For neoplasms, CDC WONDER estimates were consistently below GBD’s 95% UI lower bound, indicating a systematically larger source-dependent discordance in mortality reporting for neoplasms than for CVD.

## Discussion

This 25-year comparison reveals that although GBD and CDC WONDER depict mostly similar directional trends in CVD mortality, the magnitude of declines and increases varies between sources. Although differences in mortality estimates between the 2 databases through 2019 were previously reported,^7^ the gap widened during peak years of the COVID-19 pandemic compared with recent years. For total CVD, CDC WONDER estimates fell within GBD’s 95% UI across all years; this concordance was not observed for CVD subcategories.

The etiology of this divergence during 2020-2022 is not entirely clear but may relate to COVID-19-related disruptions to death certification practices, differences in how each system handles cause-of-death attribution for individuals with concurrent COVID-19 and pre-existing CVD, and the inherent statistical smoothing properties of GBD’s modeling framework.

Global Burden of Disease’s redistribution algorithms not only move deaths into CVD categories from ill-defined codes but also reallocate deaths initially coded to broad cardiovascular categories toward more specific non-CVD causes when covariate evidence supports such reclassification.^7^ The net effect on total CVD mortality therefore depends on the balance of inward and outward redistribution flows. Additionally, GBD has undergone iterative methodological updates across cycles that retroactively revise historical estimates,^4-6^ the impact of such revisions on observed estimates remains unclear.

The non-CVD analysis further demonstrates that database discrepancies are cause-specific in both magnitude and direction. The near-perfect concordance in all-cause mortality confirms that both systems capture a similar total number of registered deaths; observed differences therefore reflect cause redistribution rather than differential death ascertainment. Our findings indicate that CVD is not an outlier in terms of database discordance; rather, cause-specific discrepancies are an inherent feature of comparisons between administratively derived vs model-estimated mortality data.

For CVD, CDC WONDER estimates fell within GBD’s 95% UI throughout 1999-2023, suggesting broad agreement between the 2 sources regarding overall CVD mortality estimates when model uncertainty is properly accounted for; any remaining gap is consistent with different methodological approaches in GBD’s modeling. For neoplasms, the second leading cause of death in the United States, CDC WONDER estimates fell below GBD’s 95% UI lower bound in most years. This implies that the differences between the 2 data sources for neoplasm mortality extend beyond the uncertainty or confidence captured by either dataset. Researchers relying on these databases for neoplasm burden analyzes should therefore apply greater scrutiny to source selection and appreciation of methodological reporting differences during mortality reporting.

These results have important public health implications for efforts to track the effects of the pandemic on CVD mortality. For example, differences in mortality reporting between data sources may influence national public health targets, shape funding allocation decisions, and affect the ability to determine when and whether CVD mortality returns to pre-pandemic baselines. Such considerations underscore the need for careful interpretation of CVD mortality estimates across surveillance systems in the post–COVID-19 era. For policy, surveillance, and research, a nuanced understanding of how these systems differ—and why—can help ensure accurate interpretation of long-term US cardiovascular mortality trends.

## Limitations

This analysis is limited by its reliance on publicly available national mortality datasets, which may contain inherent misclassification or reporting delays. Additionally, because only CMRs were compared, other metrics, such as age-adjusted rates, may yield different insights.

## Conclusion

Estimates of US CVD mortality differ between CDC WONDER and the GBD databases, particularly during the COVID-19 pandemic. While both sources show similar overall trends, the magnitude of change varies across time, with pronounced divergence observed in the pandemic years. Our findings indicate that conclusions about national cardiovascular mortality trends depend on the data source used. Researchers and policymakers should interpret estimates cautiously, especially when assessing pandemic-era changes or evaluating progress toward public health targets. Greater transparency and harmonization across mortality surveillance systems are needed to improve comparability and ensure accurate monitoring of CVD burden in the United States.

## Use of generative AI

The authors used artificial intelligence–based tools (Paperpal), to support language editing and improve the clarity of the manuscript. These tools were not used for data analysis, interpretation, or generation of scientific content. The authors take full responsibility for the integrity and accuracy of the work.

## Supplementary Material

Web_Material_kwag153

## Data Availability

All data used in this study are publicly available. The CDC WONDER data can be accessed via the CDC WONDER online query system (https://wonder.cdc.gov/). The Global Burden of Disease 2023 study data can be accessed via the GBD Results Tool (https://vizhub.healthdata.org/gbd-results/).
