# Supplementary material for: US cardiovascular mortality before and during COVID-19: CDC WONDER vs GBD comparison
Source: Am J Epidemiol. 2026 Jul 8;195(10):2648–50. doi: 10.1093/aje/kwag153 (PMC13642362; doi:10.1093/aje/kwag153)
Supplement: Supplementary file 1 [file web_material_kwag153.docx]

**U.S. Cardiovascular Mortality Before and During COVID-19: CDC WONDER vs GBD Comparison**

**Authors:**
Mirza Mohammad Ali Baig, MBBS; Muhammad Umar, MBBS; Carol Wright Becker, MD; Salim S. Virani, MD, PhD; Dmitry Abramov, MD, MPH; Xiaoming Jia, MD; Abdul Mannan Khan Minhas, MD

**Contents**

- **Table S1. Absolute and Crude Mortality Rates per 100,000 for Cardiovascular Disease in GBD and CDC WONDER (1999–2023)**
- **Table S2. Crude Mortality Rates per 100,000 and Percent Change for Categories of Cardiovascular Disease in GBD and CDC WONDER (1999–2023)**
- **Table S3. Crude Mortality Rates per 100,000 and Percent Change for All-Cause Mortality and Selected Non-Cardiovascular Disease Categories in GBD and CDC WONDER (1999–2023)**
- **Figure S1. Comparison of All-Cause Crude Mortality Rates Between CDC WONDER and GBD (1999–2023)**
- **Figure S2. Comparison of Digestive Diseases Crude Mortality Rates Between CDC WONDER and GBD (1999–2023)**
- **Figure S3. Comparison of Neoplasms Crude Mortality Rates Between CDC WONDER and GBD (1999–2023)**
- **Figure S4. Comparison of Neurological Diseases Crude Mortality Rates Between CDC WONDER and GBD (1999–2023)**

**Table S1. Absolute and Crude Mortality Rates per 100,000 for Cardiovascular Disease in GBD and CDC WONDER (1999-2023)**

| Year | GBD Deaths | GBD CMR (95% UI) | CDC WONDER Deaths | CDC WONDER CMR (95% CI) |
| --- | --- | --- | --- | --- |
| 1999 | 918,092 | 329.77 (292.59–349.50) | 954,339 | 342.01 (341.32–342.69) |
| 2000 | 906,720 | 322.68 (285.91–342.35) | 941,526 | 334.56 (333.88–335.24) |
| 2001 | 896,083 | 315.38 (278.48–335.11) | 926,999 | 325.30 (324.64–325.96) |
| 2002 | 894,842 | 311.64 (274.43–330.87) | 923,339 | 321.02 (320.37–321.68) |
| 2003 | 880,331 | 303.72 (266.76–322.54) | 907,180 | 312.70 (312.06–313.35) |
| 2004 | 841,600 | 287.52 (252.81–305.93) | 865,863 | 295.71 (295.09–296.34) |
| 2005 | 844,292 | 285.56 (251.47–304.40) | 860,843 | 291.30 (290.69–291.92) |
| 2006 | 819,733 | 274.51 (241.49–292.85) | 827,741 | 277.41 (276.81–278.01) |
| 2007 | 804,392 | 266.82 (234.28–284.65) | 810,257 | 268.98 (268.40–269.57) |
| 2008 | 811,852 | 266.79 (233.99–284.88) | 808,525 | 265.88 (265.30–266.46) |
| 2009 | 784,782 | 255.79 (224.15–273.13) | 784,742 | 255.81 (255.24–256.37) |
| 2010 | 792,265 | 256.12 (223.64–273.90) | 784,454 | 254.08 (253.52–254.64) |
| 2011 | 797,965 | 255.91 (222.83–273.59) | 783,475 | 251.44 (250.89–252.00) |
| 2012 | 801,656 | 255.06 (221.14–272.78) | 787,431 | 250.84 (250.29–251.40) |
| 2013 | 814,380 | 257.11 (222.50–275.29) | 800,937 | 253.36 (252.80–253.91) |
| 2014 | 817,732 | 256.14 (221.32–274.13) | 807,775 | 253.33 (252.78–253.89) |
| 2015 | 844,470 | 262.34 (226.87–280.75) | 836,546 | 260.27 (259.71–260.82) |
| 2016 | 845,781 | 260.77 (226.24–279.56) | 840,678 | 260.17 (259.61–260.73) |
| 2017 | 866,224 | 265.18 (230.52–284.00) | 859,125 | 263.76 (263.20–264.32) |
| 2018 | 876,558 | 266.80 (232.21–286.14) | 868,662 | 265.51 (264.95–266.07) |
| 2019 | 864,510 | 261.83 (228.29–281.22) | 874,613 | 266.46 (265.90–267.01) |
| 2020 | 895,702 | 270.23 (240.29–288.20) | 928,741 | 281.88 (281.30–282.45) |
| 2021 | 887,640 | 267.21 (236.92–285.44) | 931,578 | 280.69 (280.12–281.26) |
| 2022 | 915,730 | 274.40 (241.02–292.83) | 941,652 | 282.53 (281.96–283.11) |
| 2023 | 900,759 | 268.55 (233.25–288.47) | 915,973 | 273.49 (272.93–274.05) |
| % Change | **-1.89%** | **-18.56%** | **-4.02%** | **-20.03%** |

*CDC WONDER indicates Centers for Disease Control and Prevention Wide-Ranging Online Data for Epidemiological Research; CMR, crude mortality rate; GBD, Global Burden of Disease; UI, uncertainty interval.*

**Table S2. Crude Mortality Rates per 100,000 and Percent Change for Categories of Cardiovascular Disease in GBD and CDC WONDER (1999-2023)**

| Year | Ischemic Heart Disease | | Stroke | | Atrial Fibrillation and Flutter | |
| --- | --- | --- | --- | --- | --- | --- |
|  | **GBD, CMR (95% UI)** | **CDC WONDER, CMR (95% CI)** | **GBD, CMR (95% UI)** | **CDC WONDER, CMR (95% CI)** | **GBD, CMR (95% UI)** | **CDC WONDER, CMR (95% CI)** |
| 1999 | 213 (190–226) | 190 (189–190) | 64 (55–69) | 60 (60–60) | 7 (6–7) | 3 (3–3) |
| 2000 | 206 (184–219) | 183 (183–184) | 63 (55–69) | 60 (59–60) | 7 (6–7) | 3 (3–3) |
| 2001 | 200 (177–212) | 176 (176–177) | 62 (53–68) | 57 (57–58) | 7 (6–8) | 3 (3–3) |
| 2002 | 196 (173–209) | 172 (171–172) | 62 (53–68) | 57 (56–57) | 7 (6–8) | 4 (3–4) |
| 2003 | 189 (167–202) | 165 (165–166) | 60 (51–65) | 54 (54–55) | 7 (6–8) | 4 (4–4) |
| 2004 | 178 (157–189) | 154 (154–155) | 56 (48–61) | 51 (51–52) | 7 (6–8) | 4 (4–4) |
| 2005 | 176 (155–187) | 151 (150–151) | 55 (47–60) | 49 (48–49) | 7 (6–8) | 4 (4–4) |
| 2006 | 167 (147–179) | 143 (142–143) | 53 (46–58) | 46 (46–46) | 7 (6–8) | 4 (4–4) |
| 2007 | 161 (141–172) | 135 (134–135) | 52 (45–57) | 45 (45–45) | 7 (6–8) | 5 (5–5) |
| 2008 | 160 (140–171) | 133 (133–134) | 52 (45–58) | 44 (44–44) | 8 (7–9) | 5 (5–5) |
| 2009 | 152 (134–163) | 126 (126–126) | 50 (43–55) | 42 (42–42) | 8 (7–9) | 5 (5–5) |
| 2010 | 151 (132–162) | 123 (123–123) | 50 (43–56) | 42 (42–42) | 8 (7–9) | 5 (5–5) |
| 2011 | 149 (131–161) | 120 (120–121) | 51 (44–56) | 41 (41–42) | 8 (7–9) | 6 (6–6) |
| 2012 | 148 (129–158) | 118 (118–119) | 51 (44–56) | 41 (41–41) | 9 (7–9) | 6 (6–6) |
| 2013 | 147 (129–159) | 117 (117–117) | 51 (44–57) | 41 (41–41) | 9 (7–10) | 7 (6–7) |
| 2014 | 145 (127–156) | 114 (114–115) | 52 (45–58) | 42 (42–42) | 9 (7–10) | 7 (7–7) |
| 2015 | 146 (128–158) | 114 (114–114) | 54 (46–60) | 44 (43–44) | 9 (8–10) | 7 (7–8) |
| 2016 | 144 (126–155) | 112 (112–113) | 55 (47–60) | 44 (44–44) | 10 (8–10) | 8 (8–8) |
| 2017 | 145 (127–156) | 112 (112–113) | 56 (48–62) | 45 (45–45) | 10 (8–11) | 8 (8–8) |
| 2018 | 146 (127–157) | 112 (111–112) | 56 (48–62) | 45 (45–45) | 10 (8–11) | 8 (8–8) |
| 2019 | 140 (122–151) | 110 (110–110) | 55 (48–61) | 46 (45–46) | 11 (9–12) | 8 (8–8) |
| 2020 | 144 (127–154) | 116 (116–117) | 57 (50–63) | 49 (48–49) | 11 (10–13) | 8 (8–8) |
| 2021 | 141 (125–151) | 113 (113–113) | 57 (49–62) | 49 (49–49) | 11 (9–13) | 8 (8–9) |
| 2022 | 144 (126–154) | 111 (111–112) | 59 (51–65) | 50 (49–50) | 12 (10–13) | 9 (9–9) |
| 2023 | 141 (123–152) | 104 (104–105) | 57 (49–63) | 49 (48–49) | 12 (10–13) | 9 (8–9) |
| % Change | **-33.9%** | **-45.0%** | **-11.1%** | **-19.0%** | **+74.1%** | **+186.0%** |

*CDC WONDER indicates Centers for Disease Control and Prevention Wide-Ranging Online Data for Epidemiological Research; CMR, crude mortality rate; GBD, Global Burden of Disease; and UI, uncertainty interval.*

**Table S3. Crude Mortality Rates per 100,000 and Percent Change for All Cause Mortality and Selected Non-Cardiovascular Disease Categories in GBD and CDC WONDER (1999-2023)**

| Year | All-Cause Mortality | | Digestive Diseases | | Neoplasms | | Neurological Diseases | |
| --- | --- | --- | --- | --- | --- | --- | --- | --- |
|  | **GBD, CMR (95% UI)** | **CDC WONDER, CMR (95% CI)** | **GBD, CMR (95% UI)** | **CDC WONDER, CMR (95% CI)** | **GBD, CMR (95% UI)** | **CDC WONDER, CMR (95% CI)** | **GBD, CMR (95% UI)** | **CDC WONDER, CMR (95% CI)** |
| 1999 | 859 (858–860) | 857 (856–858) | 32 (29–35) | 29.7 (29.5–29.9) | 218 (203–226) | 202 (201–202) | 58 (24–126) | 30.1 (29.9–30.3) |
| 2000 | 855 (854–856) | 854 (853–855) | 33 (30–35) | 29.9 (29.7–30.1) | 217 (203–225) | 201 (201–202) | 59 (25–127) | 32.3 (32.1–32.6) |
| 2001 | 850 (850–851) | 848 (847–849) | 33 (30–36) | 30.1 (29.9–30.3) | 215 (201–223) | 199 (199–200) | 59 (25–128) | 33.9 (33.7–34.1) |
| 2002 | 851 (850–852) | 850 (848–851) | 34 (30–36) | 30.2 (30.0–30.4) | 215 (201–223) | 198 (198–199) | 60 (26–130) | 35.8 (35.6–36.1) |
| 2003 | 844 (844–845) | 844 (843–845) | 34 (30–36) | 30.3 (30.1–30.5) | 213 (200–221) | 197 (196–197) | 61 (26–131) | 37.8 (37.6–38.0) |
| 2004 | 820 (819–820) | 819 (818–820) | 33 (30–36) | 29.5 (29.3–29.7) | 209 (196–218) | 194 (193–194) | 61 (26–129) | 38.2 (37.9–38.4) |
| 2005 | 828 (827–829) | 828 (827–829) | 34 (30–36) | 29.6 (29.4–29.8) | 210 (196–219) | 194 (193–194) | 63 (27–133) | 40.9 (40.6–41.1) |
| 2006 | 813 (812–813) | 813 (812–814) | 33 (30–36) | 29.4 (29.2–29.6) | 208 (194–217) | 192 (192–193) | 62 (27–132) | 41.2 (41.0–41.4) |
| 2007 | 804 (803–805) | 805 (804–806) | 34 (31–36) | 29.6 (29.4–29.8) | 207 (192–217) | 192 (191–192) | 63 (27–133) | 42.2 (41.9–42.4) |
| 2008 | 812 (812–813) | 813 (812–814) | 34 (32–37) | 30.0 (29.8–30.2) | 210 (194–219) | 191 (190–191) | 65 (28–137) | 44.9 (44.7–45.2) |
| 2009 | 794 (794–795) | 794 (793–795) | 34 (31–37) | 29.2 (29.0–29.3) | 208 (192–217) | 190 (189–190) | 65 (28–135) | 43.7 (43.5–44.0) |
| 2010 | 799 (798–799) | 800 (799–801) | 35 (32–37) | 29.6 (29.4–29.8) | 208 (191–217) | 191 (191–191) | 66 (29–139) | 45.7 (45.5–45.9) |
| 2011 | 807 (806–807) | 807 (806–808) | 36 (32–38) | 30.2 (30.0–30.4) | 209 (191–218) | 190 (189–190) | 68 (30–141) | 46.5 (46.3–46.8) |
| 2012 | 809 (808–810) | 810 (809–811) | 36 (33–39) | 30.4 (30.2–30.6) | 209 (191–219) | 190 (190–191) | 69 (30–143) | 46.6 (46.3–46.8) |
| 2013 | 820 (819–820) | 822 (821–823) | 37 (33–40) | 31.1 (30.9–31.3) | 210 (193–220) | 190 (189–190) | 70 (31–145) | 48.0 (47.8–48.3) |
| 2014 | 823 (822–824) | 824 (823–825) | 37 (33–40) | 31.7 (31.5–31.9) | 210 (193–220) | 191 (190–191) | 71 (32–146) | 51.9 (51.6–52.1) |
| 2015 | 843 (842–843) | 844 (843–845) | 38 (34–41) | 32.6 (32.4–32.8) | 211 (194–221) | 190 (190–191) | 73 (33–150) | 59.3 (59.0–59.5) |
| 2016 | 846 (845–847) | 849 (848–850) | 38 (34–41) | 32.8 (32.6–33.0) | 210 (193–220) | 190 (189–190) | 74 (33–150) | 62.6 (62.4–62.9) |
| 2017 | 861 (860–862) | 864 (863–865) | 38 (34–41) | 33.1 (32.9–33.3) | 210 (193–221) | 189 (188–189) | 76 (35–155) | 66.4 (66.1–66.7) |
| 2018 | 864 (863–865) | 868 (867–869) | 38 (34–41) | 33.6 (33.4–33.8) | 208 (191–218) | 188 (187–188) | 77 (35–156) | 68.8 (68.5–69.0) |
| 2019 | 865 (864–866) | 870 (869–871) | 39 (35–42) | 34.4 (34.2–34.6) | 208 (190–218) | 187 (187–188) | 79 (37–159) | 71.2 (70.9–71.5) |
| 2020 | 1021 (1020–1021) | 1027 (1026–1028) | 41 (37–44) | 37.6 (37.4–37.8) | 211 (196–220) | 188 (187–188) | 81 (38–166) | 79.6 (79.3–79.9) |
| 2021 | 1043 (1042–1043) | 1044 (1043–1045) | 41 (38–45) | 40.3 (40.1–40.6) | 210 (194–219) | 187 (187–188) | 82 (38–166) | 75.8 (75.5–76.1) |
| 2022 | 983 (982–984) | 984 (983–985) | 42 (38–45) | 39.8 (39.6–40.0) | 212 (196–222) | 187 (187–188) | 84 (40–168) | 79.3 (79.0–79.7) |
| 2023 | 922 (921–922) | 923 (922–924) | 41 (37–45) | 38.4 (38.1–38.6) | 211 (193–222) | 188 (187–188) | 84 (40–169) | 78.7 (78.4–79.0) |
| % Change | +7.3% | +7.7% | +29.0% | +29.1% | –3.3% | –6.9% | +44.4% | +161.3% |

*CDC WONDER indicates Centers for Disease Control and Prevention Wide-Ranging Online Data for Epidemiological Research; CMR, crude mortality rate; GBD, Global Burden of Disease; and UI, uncertainty interval.*

**Figure S1. Comparison of All-Cause Crude Mortality Rates (CMR) per 100,000 Population Between CDC WONDER and GBD (1999–2023).**

The figure displays annual CMR estimates from CDC WONDER (solid line with 95% confidence intervals [CIs], shaded blue) and GBD (solid line with 95% uncertainty intervals [UIs], shaded red) for all-cause mortality.

**Alt Text:** Line graph comparing all-cause mortality rates from CDC and GBD data between 1999 and 2023, with crude mortality rates and confidence/Uncertainty intervals shown in blue for CDC and red for GBD.

**Figure S2. Comparison of All Digestive Diseases Crude Mortality Rates (CMR) per 100,000 Population Between CDC WONDER and GBD (1999–2023).**

The figure displays annual CMR estimates from CDC WONDER (solid line with 95% confidence intervals [CIs], shaded blue) and GBD (solid line with 95% uncertainty intervals [UIs], shaded red) for all digestive diseases.

Alt Text: Line graph comparing digestive diseases mortality rates from CDC and GBD data between 1999 and 2023, with crude mortality rates per 100,000 population on the y-axis and years on the x-axis. The graph features two sets of lines with shaded 95% confidence/uncertainty intervals in blue for CDC and red for GBD.

**Figure S3. Comparison of All Neoplasms Crude Mortality Rates (CMR) per 100,000 Population Between CDC WONDER and GBD (1999–2023).**

The figure displays annual CMR estimates from CDC WONDER (solid line with 95% confidence intervals [CIs], shaded blue) and GBD (solid line with 95% uncertainty intervals [UIs], shaded red) for all neoplasms.

**Alt Text:** Line graph comparing neoplasms mortality rates from CDC and GBD data between 1999 and 2023, with crude mortality rates and confidence/uncertainty intervals shown. CDC rates, depicted in blue with solid lines and shaded confidence intervals, show a decreasing trend, while GBD rates in red with dashed lines and wider uncertainty intervals remain higher and more variable.

**Figure S4. Comparison of All Neurological Diseases Crude Mortality Rates (CMR) per 100,000 Population Between CDC WONDER and GBD (1999–2023).**

The figure displays annual CMR estimates from CDC WONDER (solid line with 95% confidence intervals [CIs], shaded blue) and GBD (solid line with 95% uncertainty intervals [UIs], shaded red) for all neurological diseases.

**Alt Text:** Line graph comparing neurological disorders mortality rates from CDC and GBD data between 1999 and 2023, showing an overall increasing trend. The chart includes two lines with confidence/uncertainty intervals shaded in blue for CDC and red for GBD, highlighting a rising gap in mortality rates over time.
